# Global Assessment of Emerging Contaminant Removal in Wastewater Treatment Plants: *In Silico* Hazard Screening and Risk Evaluation

**DOI:** 10.3390/toxics13010006

**Published:** 2024-12-25

**Authors:** Arianna Sgariboldi, Elena Posté, Nicola Chirico, Alessandro Sangion, Marco Evangelista, Cristiana Morosini, Andrea Re, Vincenzo Torretta, Ester Papa

**Affiliations:** 1 Department of Theoretical and Applied Sciences, University of Insubria, via J.H. Dunant 3, 21100 Varese, Italy; asgariboldi@uninsubria.it (A.S.);; 2 Department of Science and High Technology, University of Insubria, via Valleggio 11, 22100 Como, Italy; 3 AIR CLEAN S.r.l., via Trento 37, 20017 Rho, Italy; 4 ARC Arnot Research & Consulting, Toronto, ON M4M-1W4, Canada; 5 Xylem Water Solutions Italia S.r.l., Via G. Rossini, 1/A, 20045 Lainate, Italy

**Keywords:** pharmaceuticals and personal care products, QSAR, PMT, PBT, new approach methodologies, risk assessment, *in silico* tools

## Abstract

Pharmaceuticals and personal care products (PPCPs) are emerging contaminants (ECs), whose presence in the environment is of increasing concern due to their widespread use and possible detrimental effects on wildlife and humans. These chemicals may present multiple hazardous properties such as environmental persistence, toxicity, high mobility, and the potential for bioaccumulation. In this study, extended bibliographic research was conducted to characterize the removal efficiency (RE) of PPCPs in wastewater treatment plants (WWTPs) considering different technologies. Measured values of RE were collected from the literature or calculated for 251 compounds. The molecular structure of the 245 PPCPs were used as the input to generate predictions of multiple properties using several QSAR tools, such as the OECD Toolbox, OPERA, EPI Suite™, and QSAR-ME Profiler. These predictions were compared to regulatory thresholds to identify hazardous chemicals and to screen persistent, mobile and toxic (PMT) or persistent, bioaccumulative and toxic (PBT) substances. Finally, chemicals were prioritized by combining values of RE and QSAR predictions for multiple properties. A total of 16 out of the 245 molecules were prioritized as the most hazardous compounds to the aquatic environment and, among these, six were associated with potential risk due to their exposure concentrations reported in the literature.

## 1. Introduction

Pharmaceuticals and personal care products (PPCPs) are emerging contaminants (ECs) [1,2,3], whose presence in the environment is of increasing concern. PPCPs belong to a wide variety of chemical classes and are generally biologically active substances whose effects can be observed at low concentrations [4]. These substances may cause adverse effects on humans and the environment [1,5,6,7]. Several studies show that the uncontrolled release of PPCPs is associated with adverse effects in various trophic levels of the aquatic environment, where non-target organisms are exposed to environmental concentrations of PPCP residues. These exposures result in both acute and chronic effects [8], including sexual dysfunction [9], feminization of male fish [10], fish physiology and behavior alteration [11], inhibition of the regeneration ability [12], alteration of cellular responses in liver, kidney and gills [13], and endocrine disruption (ED) [5,14,15,16].

PPCPs are typically released into the environment through continuous emissions at low concentrations and tend to be ubiquitous. In fact, all the environmental compartments are contaminated by PPCPs, with concentrations ranging from ng/L to μg/L [17], and human and veterinary consumption are the main sources responsible for their presence. Recent research [18,19,20] highlighted several pathways as the most responsible for the release of PPCPs, such as leakage from sewers and uncontrolled landfills, usage of treated wastewater for irrigation and sewage sludge used as fertilizer by spreading over agricultural soil, veterinary and aquaculture use, domestic use through human daily washing activities, industrial effluents, hospital effluents, and septic tanks. Evidence of PPCPs in natural and drinking waters [21] also suggests that they are not efficiently removed by wastewater treatment plants (WWTPs) [18,20].

In the recent years, several studies have been conducted that highlight potential hazardous properties of PPCPs, such as persistence, bioaccumulation, toxicity, and mobility in water [22,23,24,25,26,27]. Persistent, bioaccumulative and toxic (PBT) substances, as well as very persistent and very bioaccumulative (vPvB) substances are among the most hazardous categories of interest for the academic, industrial, and regulatory fields. In Europe these substances are governed by the REACH Regulation, and the criteria for their identification are detailed in its Annex XIII [28,29]. Furthermore, the recent European delegated regulation on classification, labeling, and packaging of chemicals (CLP) EU 2023/707 [30] has introduced two new classes of hazardous substances identified as persistent, mobile and toxic (PMTs) and very persistent and very mobile (vPvM), which need to be properly managed to guarantee the safety of natural environments and drinking waters [31,32]. These classes of hazardous substances were added to those already included in the regulation EU 1272/2008 [33].

The regulatory requirements [28,30,31,32,33] to identify and screen potentially hazardous chemicals such as PBTs and PMTs, while minimizing the experimental costs, prompts the use of *in silico* approaches, for instance models based on quantitative structure–activity relationships (QSARs) [34,35]. These models can predict multiple properties and activities of heterogeneous, new and existing, chemicals from their molecular structure to identify safer alternatives to problematic substances [26,36,37].

This work is based on an extended research of the literature, specifically studies published between 2014 and 2019, which was performed in a former academic study [38] to quantify, in terms of removal efficiency (RE), the resistance to degradation of PPCPs released from several WWTPs worldwide. Starting from that valuable study, which included thousands of curated data entries, the first aim of this work is to focus on the in-depth analysis of the RE profile of more than 200 PPCPs, based on different WWTPs technologies in five continents.

The second aim is to identify the PPCPs of greatest concern by predicting their potential hazard from the molecular structure using different *in silico* tools such as EPI Suite™ v.4.1 [39], Open [Quantitative] Structure-activity/property Relationship App [OPERA] v 2.9 [40], QSAR Toolbox v. 4.4 [41], and QSAR-ME v. 1.02 [42], and focusing on their potential PMT and PBT behavior.

The final aim of this work is to combine experimental RE and environmental concentrations reported in the literature with the *in silico* predictions, to perform a PMT and PBT assessment, and identify those PPCPs that may pose a risk for the aquatic environment.

We want to highlight that the focus of this study is to demonstrate how the integration of experimental and *in silico* data is useful at the screening level to spot potentially hazardous compounds or possible risk situations. This was performed here using data published between 2014 and 2019, not to ignore the current reality but to maximize the value of former, laboriously collected data. The proposed methodology is adaptable to the rapidly changing environment as it can be updated using new data for concentrations and RE and is flexible to the inclusion of new *in silico* models of improved quality.

## 2. Materials and Methods

### 2.1. Bibliographic Selection

The literature was explored using ISI Web of Science [43] and Google Scholar [44], considering articles published between 2014 and 2019, using the following keywords listed in alphabetic order: “activated sludge”, “emerging contaminants”, “hydraulic retention time”, “illicit drugs”, “municipal wastewater”, “pharmaceuticals”, “pharmaceuticals removal”, “PPCPs”, “personal care products”, “removal”, “removal efficiency”, “sewage”, “wastewater”, “wastewater treatment plant”, “water treatment”, and “WWTP”. Only articles reporting information on at least 5 chemicals were considered. A total of 73 manuscripts were initially selected with these criteria. Among these, only manuscripts including information on WWTPs, as well as the quantification of RE or concentrations of the chemicals in influents and effluents (used to calculate RE), were included in the final study. Data without any specification regarding the WWTPs, i.e., data not reporting values for all the samples of all the WWTPs, were discarded from the analysis. The final list of the 32 articles used in this study is reported in Supplementary Material S1—Table S1.

### 2.2. Creation of the Dataset and Data Curation

Information regarding chemical identifiers (i.e., name, Chemical Abstract Service registry number [CAS], and Simplified Molecular Input Line Entry System [SMILES]), WWTPs characterization, RE, sampling, and sorption to sludge, was initially collected for 251 chemicals. A total of 2034 records were extracted from the selected publications, with each record containing chemical information and a consistent set of data from a specific source (Supplementary Material S1—Table S2).

#### 2.2.1. Creation of the Quality Index

To extract relevant data from Supplementary Material S1—Table S2 and select appropriate RE values and chemicals for the analysis, we created three quality indices. These indices were designed to categorize, standardize, and compare the available literature information on chemical identifiers (molecule identification index), RE quantification (RE data index), and WWTP process characteristics (general information index). These indices are described in detail in Supplementary Material S2 and their values are reported in Supplementary Material S1—Table S2. The combination of the indices (i.e., the sum of the scores of the individual indices) led to the quality index (QI) which was used to assign each record to four quality classes as follows:

- score 0–1 = Quality index low (QI Low);
- score 2–3 = Quality index moderate (QI Moderate);
- score 4–5 = Quality index good (QI Good);
- score 6 = Quality index excellent (QI Excellent).

Records assigned to QI Low were excluded from further analysis, which led to the exclusion of 6 chemicals from the initial dataset.

#### 2.2.2. Characterization of the WWTPs

Starting from the information collected in the literature (Table S1), WWTPs were classified as WWTP1 or WWTP2 based on the traditional levels of treatment reported in Table 1 (when clearly indicated), or on the treatment technologies described in Table 1.

However, in the presence of dubious situations, when the level of treatment in the literature was not clearly indicated, and only one method for tertiary/advanced treatment was reported, the classification given was WWTP1 following the worst-case approach. The final classification and the information included in each study characterizing different WWTPs is reported in Supplementary Material S1—Table S1.

#### 2.2.3. Removal Efficiency Dataset

After the exclusion of records classified as QI Low, RE was quantified for each remaining chemical using information from the literature. When not reported in the literature, RE was obtained using different extrapolations or calculations, depending on the availability of data related to specific factors such as hydraulic retention time (HRT) or the sludge retention time (SRT) [4,8,46,47,48].

When RE was calculated in the original literature, it was possible, in most cases, to understand which data and/or statistics were used, e.g., median, average, mass load, etc. However, when the RE was missing, it was calculated using the median values of the concentrations from influents and effluents. Median, if available, was preferred over average because it is less sensitive to extreme values.

The equation used to calculate the RE was:

(1) $$RE\left[\%\right]=1-\frac{c_{effl}}{c_{infl}}\cdot 100$$

where:

*c_effl_* is the concentration of the compound in the effluent,

*c_infl_* is the concentration of the compound in the influent.

In some papers, the authors provided more complex equations. For example, [8] proposed the following:

(2) $$RE\;[\%]=\frac{\left[C_{i}\cdot F\right]-[\left(C_{e}\cdot F\right)+(C_{s}\cdot TSP)]}{\left[C_{i}\cdot F\right]}\cdot 100$$

where:

*C_i_* is the mean concentration of the analyte in wastewater influent (ng/L),

*C_e_* is the mean concentration of the analyte in wastewater effluent (ng/L),

*C_s_* is the mean concentration of the analyte in sludge (ng/g wet weight),

*F* is the daily flow of wastewater influent (L/d),

*TSP* is the total sludge production (g/d wet weight).

When all values of the parameters were available, but the REs were missing, Equation (2) was used instead of Equation (1).

The full list of chemicals and the raw RE data (collected or calculated) are listed in Supplementary Material S1—Table S2.

It is necessary to highlight that the previous equations could not be used in certain specific situations, for example, when the compound was not detected in the influent and/or in the effluent sample or when some data were missing. Table S1 in Supplementary Material S2 describes the evaluation criteria adopted to deal consistently with such specific situations. Furthermore, negative RE was also available for some compounds. This may be ascribed to the de-conjunction of excreted conjugated forms or to the transformation of parental compounds into their metabolites within the WWTPs [1,49,50,51,52]. RE final data were ranked according to six classes (i.e., “other”, “low”, “moderate”, “high”, “excellent” and “no data”) which were attributed based on the criteria described in Table 2.

Chemicals with RE Low or Other were considered as resistant to treatment, applying a conservative (worst-case) approach.

Finally, a consensus approach was applied when multiple RE classes were available for a single chemical by assigning each compound to the most represented RE class, considering the frequency of each class. Consensus RE classes were assigned to keep the data for WWTP1 and WWTP2 separate. When the classes had the same number of records, the worst-case class was assigned (worst-case consensus). When the most populated class was “no data”, the next class with the major number of records was assigned.

The RE classes assigned for single chemicals, after consensus analysis, are listed in Supplementary Material S1—Table S3.

#### 2.2.4. Chemical Dataset

After elimination of those records classified as QI Low, further data curation was performed on data included in Supplementary Material S1—Table S2 to harmonize, complete, and correct, if necessary, the information retrieved for the chemicals found in the selected articles listed in Supplementary Material S1—Table S1. When a chemical identifier (i.e., CAS, Name or SMILES) was missing or unclear, it was cross-checked using the PubChem chemical database [53] and the commercial websites of the suppliers of the analytical standards. This search was carried out by name or, when possible, by CAS number. Two salts were converted into the neutral form of the respective drug when QSAR analysis was performed.

Finally, SMILES were converted to canonical forms using Open Babel ver. 2.4.1 [54]. The list of the final 245 unique molecules (after elimination of QI-Low records), together with their CAS, name, and canonical SMILES, is given in Supplementary Material S1—Table S3.

### 2.3. QSAR Predictions

In recent years, several studies [37,55,56] and regulations [30,57] have suggested how to identify PBT and PMT chemicals through screening analysis performed by QSAR models. Table 3 lists the criteria used in this study to identify PMT or PBT substances among those listed in Supplementary Material S1—Table S3, using QSAR predictions based on different freely available software commonly applied for regulatory purposes (EPI Suite^TM^ v. 4.1 [39], OPERA v. 2.9 [40], OECD QSAR Toolbox v. 4.4 [41], and QSAR-ME-Profiler v. 1.02 [42]). In particular, Table 3 includes regulatory screening thresholds used at the European level for the compilation of regulatory dossiers, suggested by the German Environment Agency (Umweltbundesamt—UBA) in 2019 [31], by the European Commission in 2021 [30,57], as well as those reported in the literature [36,37].

In addition, criteria to screen potential endocrine disruptors (EDs) have been included in the evaluation. In fact, endocrine disruption has been recently added as a new hazard class in the delegated regulation on classification, labeling, and packaging of chemicals (CLP) [30]. Moreover, additional models for non-regulatory endpoints have been applied to provide further information to support the PBT screening.

Two levels of screening were used in this study to assess the potentially hazardous PMT or PBT behavior of chemicals. The first (Table 4—Screening Level L1) was based on endpoints and QSAR models which were selected according to regulatory requirements. The second (Table 4—Screening Level L2) was meant to provide additional information, using alternative endpoints and QSARs [62,63,64] not suggested at the regulatory level.

As mentioned before, different *in silico* tools were applied to predict PMT, PBT, and ED properties by QSAR. This work was focused on freely available software, commonly used for regulatory purposes (i.e., EPI Suite^TM^ v. 4.1, OECD QSAR Toolbox v. 4.4, and OPERA v. 2.9), in addition to in-house software, recently proposed and freely distributed by our research group (QSAR-ME Profiler v.1.02). Only predictions included in the applicability domain (AD) of the respective model [35] were used for further analysis. Information on the AD was provided by the software OPERA v. 2.9 [65] and QSAR-ME Profiler v. 1.02 [42]. The AD of BIOWIN, KOWWIN, and KOCWIN models, available in EPI Suite™ v. 4.1 and in the OECD QSAR Toolbox v. 4.4, was calculated using in-house R scripts [66] following a similar procedure as the one recently illustrated by Zhang and colleagues [67]. This was necessary as no clear indication on the inclusion of predictions within the models AD was provided by EPI Suite™.

It is necessary to highlight that the EPI Suite™ KOWWIN and KOCWIN (from the molecular connectivity index (MCI)) models provide a predicted value in addition to the experimental value, when available. Therefore, the evaluation of the B and M properties was performed by giving priority to experimental data provided by EPI Suite™ v. 4.1. However, when experimental octanol–water partitioning ratio (Kow) or organic carbon–water partitioning ratio (Koc) (in base 10 logarithmic form [Log]) values were not available, predictions within the AD of the models listed in Table 4, referred to as “L1-Regulatory”, were averaged and used for the evaluation of B and M. Furthermore, for Log Koc predictions, the model “Soil Koc” [68] was selected when the predictions from the “KOCWIN from MCI” model were found to be out of the AD (correlation among reliable predictions generated by Soil Koc and KOCWIN (MCI) = 0.85).

The evaluation of T was performed using QSAR predictions calculated for three trophic levels (i.e., algae, *Daphnia,* and fish). This information was used in Section 3.4 for the evaluation of the potential risk associated to the studied PPCPs.

It is important to highlight that the PPCPs dataset contains 233 ionizable organic chemicals (IOC) that might be present in a dissociated form depending on the pH of the medium. Unfortunately, there is currently a lack of QSAR tools for IOCs and the models used in this screening exercise were mainly developed on neutral organic chemicals. We used a neutralized version of the SMILES (i.e., adding a proton) to obtain predictions that must be considered only for the neutral form of the chemical.

**Figure 1:**
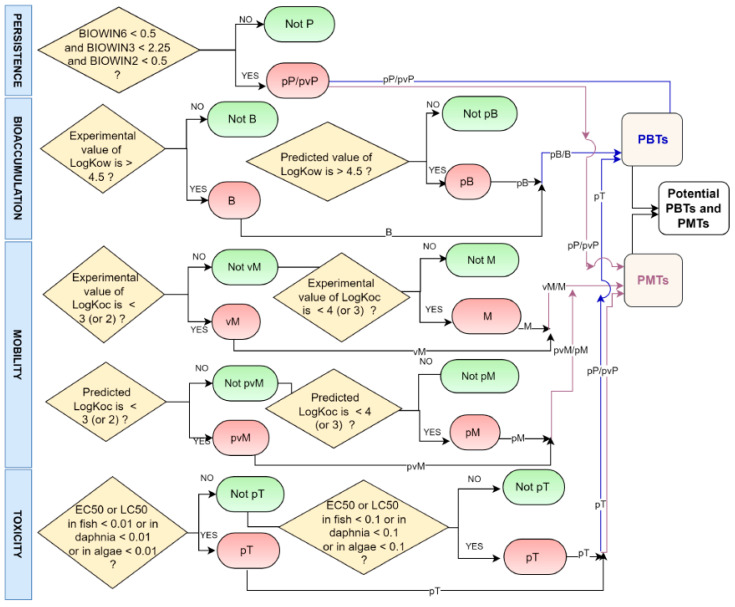
Schematic workflow used in this study to screen PMT/PBT chemicals using regulatory criteria as reported in Table 3.

The procedure followed to screen PMT and PBT chemicals, according to criteria specified in Table 3 and using values provided by the tools listed in Table 4, is schematized in Figure 1.

Furthermore, the potential ED behavior of the studied chemicals was performed using QSAR models to predict the binding to the human transthyretin (hTTR, a thyroid hormone distributor protein), estrogen, and androgen receptors [59,60,61,69,70]. The list of tools used for ED screening is shown in Table 5.

### 2.4. Potential Risk Evaluation

The potential risk (PR), associated to PPCPs included in Supplementary Material S1—Table S2 (i.e., excluding the data with QI low), was quantified for the aquatic environment according to the technical guidance document (TGD) [71], based on measured concentrations in the WWTPs effluents, and predicted acute toxicity (Lethal or Effect concentrations to 50% of the test animals, respectively, LC_50_ and EC_50_) in three trophic levels (i.e., algae, *Daphnia*, and fish) and considering a worst-case scenario. The following formula was applied separately for LC_50_ and EC_50_ data

Potential Risk (PR) = CA/PNEC > 1(3)

and predicted no–effect-concentrations (PNECs) were calculated for each chemical and each measure of aquatic concentration by dividing the minimum values of LC_50_ or EC_50_, among those predicted using QSARs across the different trophic levels [71], by an assessment factor of 1000. CA is the concentration measured for a chemical in the WWTP effluent. CA values were extracted from references listed in Supplementary Material S1—Table S1 and are reported in Supplementary Material S1—Table S2. Chemicals with no measured CA were excluded from the quantification of the PR. All the predicted values of LC_50_ or EC_50_, as well as CA data, were converted to ng/L for homogeneity. Multiple values of PR were calculated for chemicals with CA measured in different WWTPs, furthermore, PR was evaluated separately for predicted values of LC_50_ or EC_50_. Finally, a compound was highlighted for its PR when at least one among the calculated PR was >1.

The list of chemicals and the result of the prioritization according to PR are reported in Supplementary Material S1—Table S2.

## 3. Results and Discussion

### 3.1. Data Collection and Curation

The data collected from the 32 papers listed in Supplementary Material S1—Table S1 led to a final dataset including 2034 records for 251 molecules listed in Supplementary Material S1—Table S2. The quality analysis of these records, based on the QI described in Section 2.2.1, highlighted 282 records (13.86%) with QI Low, 940 records (46.21%) with QI Moderate, 794 records (39.04%) with QI Good, and 18 records (0.88%) with QI Excellent (Supplementary Material S1—Table S2). It is interesting to highlight that only 14% of the records were associated with low quality information, i.e., information which was insufficient to provide a good characterization of the molecular structure or to quantify the RE, or missed other information needed to quantify RE or to characterize the WWTP. However, records of moderate to excellent quality were available for most of the chemicals, so low quality records were removed from the dataset and discarded from further analysis, leading to the exclusion of six chemicals from the dataset. The 245 chemicals remaining after cleaning the dataset, are listed in Supplementary Material S1—Table S3.

**Figure 2:**
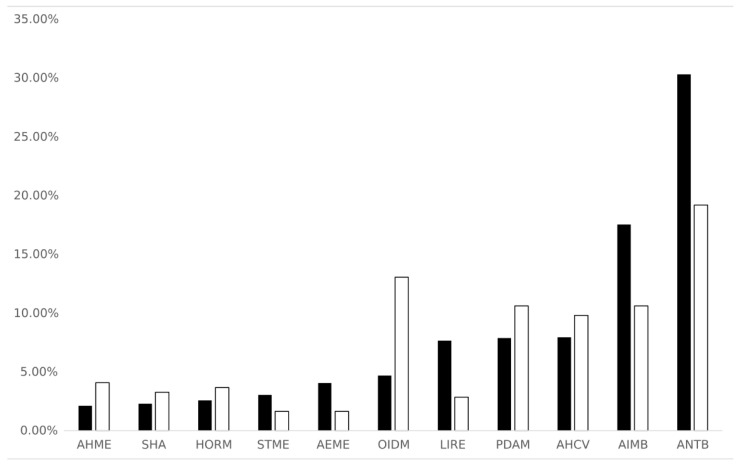
Classes of molecules and related percentages of molecules and records (greater than 3%) (white bars = % of molecules; black bars = % of records). Acronyms are described in Supplementary Material S2—Table S4.

Figure 2, Figure S1 in Supplementary Material S2, and Table S3 in Supplementary Material S2 show that PPCPs included in this study are representative of multiple classes of use, among these are, antibiotics (ANTB) (47 chemicals), opioids, illicit drugs and metabolites (OIDM) (both illicit and prescribed) (32 chemicals), psychiatric drugs/antidepressants and metabolites (PDAM), and analgesics/anti-inflammatories and metabolites (AIMB) (26 chemicals) which are the most populated classes when considering the number of molecules per class. Among these, ANTB, AIMB, and PDAM are also the classes with the largest number of RE records (i.e., 531, 307, and 139, respectively).

### 3.2. WWTPs Characterization and RE Analysis

In total, 76 WWTPs were analyzed in this study: thirty-six (47.37%) in Europe, twenty-eight (36.84%) in Asia, ten (13.16%) in America, one (1.32%) in Africa, and one (1.32%) in Oceania.

**Figure 3:**
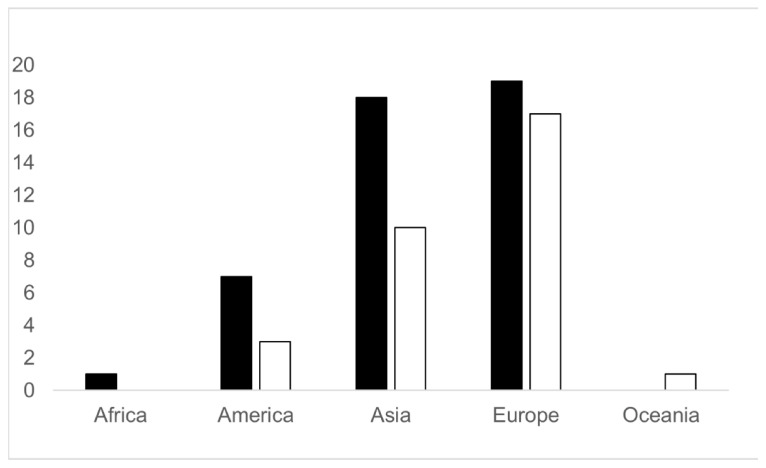
Worldwide distribution of the studied WWTPs, divided on the basis of the treatment (black bars = WWTP1; white bars = WWTP2). The number of plants analyzed is reported on the y axis.

Globally, 59% of the analyzed WWTPs were classified as WWTP1, while the remaining 41% classified as WWTP2. Figure 3 shows that the studied WWTPs are mostly located in Europe and Asia. In Europe, approximately half of the analyzed plants are WWTP2, compared to one third of the WWTPs in Asia and America classified as WWTP2.

Regarding the 245 chemicals identified from the literature studies, 224 were detected in the effluents of WWTP1 and 109 in the effluents of WWTP2. Each record was assigned to a class of RE and the consensus approach was applied in the presence of multiple RE data and classes, as described in the methods section (RE data are reported in Supplementary Material S1—Tables S2 and S3). Table 6 shows that WWTP1 are about 50% less efficient than WWTP2 considering PPCPs characterized by low RE or Other. On the other hand, in total, about 60% of the chemicals have moderate to excellent RE in WWTP2, while only about 40% has moderate to excellent RE in WWTP1.

More in detail, considering the 103 PPCPs identified as the most resistant to treatment in WWTP1 effluents, i.e., those more frequently assigned to Low RE and Other RE, 18 were ANTB, 18 were PDAM, 12 were AHCV and 10 were AIMB. Differently, among the most abundant PPCPs found in WWTP2 effluents, six were ANTB, six were OIDM and five were SHA.

**Figure 4:**
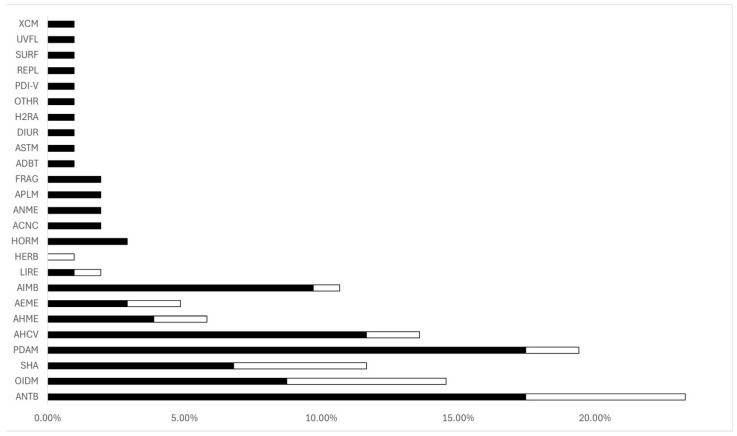
Cumulative percentages of PPCPs resistant to treatment (i.e., Low and Other RE) in WWTP1 (black bars) and WWTP2 (white bars). Acronyms are described in Supplementary Material S2—Table S4.

Histograms in Figure 4 clearly show that ANTB, which are the most abundant group of PPCPs measured in effluents of both WWTP1 and WWTP2 with PDAM, OIDM, AHCV, SHA, and AIMB are less efficiently removed mostly in WWTP1. Considering the less abundant classes in WWTPs effluents, they are mostly not removed efficiently by WWTP1.

To explore WWTPs efficiency for individual compounds, only chemicals with RE records quantified for both WWTP1 and WWTP2 were further analyzed (i.e., 71 compounds). Table S4 in Supplementary Material S1 shows that in most cases, as expected, the RE of chemicals is equal or improves with the level of treatment, moving from WWTP1 to WWTP2. However, there are some exceptions (i.e., diphenhydramine, sulfamethoxazole, alprazolam, sotalol, diazepam, ampicillin, and sulfapyridine) which are largely explainable considering that, as was mentioned in Section 2.2.2, RE classes were assigned to the most represented RE class among those available, or, in case of equality in the numbers of data available for each RE class, to the worst class. For instance, sotalol had one record with RE Low in WWTP1 and two records in WWTP2 (one record with RE Low and another with RE Other), therefore it was assigned to the RE Other class for WWTP2. Ampicillin was classified as RE High in WWTP1 and as RE Other in WWTP2, according to the worst-case approach. Chloramphenicol was assigned to the class RE Moderate in WWTP1, but to RE Other in WWTP2. However, it is interesting to highlight that literature studies have documented the formation of this compound after advanced treatment. Similarly, the literature studies [6,52,72,73,74] reported the formation of diclofenac, lorazepam, methadone, metoprolol, and propranolol after advanced treatment. Additional details about the aforementioned exceptions are reported in Supplementary Material S2—Table S5.

Regarding substances classified as RE Other in both WWTP1 and WWTP2, the literature shows that some substances can form during the treatment process due to the characteristics of the plant and of the chemical itself [51,52,73,74,75]. Examples include carbamazepine, erythromycin [51], norcocaine [49,73,74], oxazepam, and venlafaxine [52]. Other substances, such as the already mentioned lorazepam, methadone, metoprolol, and propranolol, in addition to methamphetamine, sulpiride, and nalidixic acid, tend to reconstitute when they are treated with secondary treatments only [6,52].

Finally, the analysis of Tables S2 and S4 in Supplementary Material S1 reveals that the substances resistant to treatment in WWTP1, but not in WWTP2 (i.e., sulpiride, nalidixic acid, clindamycin, methamphetamine, roxithromycin, sertraline, clarithromycin, clofibric acid, fluoxetine, lincomycin, ranitidine, and trimethoprim), have been detected in developing countries such as Africa and/or Asia. Furthermore, in this study we had no records measured in a WWTP2 in Africa and detected a lower number of WWTP2 than WWTP1 in Asia (Figure 3). This highlights the need for investment in these regions to improve treatment plants (e.g., to increase the treatment level from secondary to advanced) or to replace substances recalcitrant to degradation, most of which are pharmaceuticals, with alternatives that are easily removed by secondary treatment plants.

### 3.3. In Silico Screening of PBTs and PMTs

A battery of 24 QSAR models listed in Table 3 were applied to screen PMT and PBT properties in addition to ED behavior for the 245 PPCPs included in dataset Table S3 in Supplementary Material S1. QSAR predictions and the AD evaluated for the different models (where available) are reported in Supplementary Material S1—Table S3.

The application of regulatory thresholds, listed in Table 3, to reliable predictions listed in Supplementary Material S1—Table S3, led to the identification of 16 potentially hazardous substances for their potential PMT or PBT behavior.

These 16 substances include ANTB (4/16), PDAM (6/16), OIDM (2/16). This result is consistent with the fact that these pharmaceutical classes were highlighted before as the most resistant to treatment in terms of RE (Figure 4) and therefore more easily available in the environment to exert their PMT and PBT behavior. This is also confirmed by RE classes reported for the chemicals listed in Table 7, except for chloramphenicol and sertraline, which instead are removed by water treatment and therefore should not represent a problem after WWTPs.

Moreover, as no RE data of sufficient quality were available for olanzapine and sulfachlorpyridazine, we considered them as resistant to treatment based on BIOWIN predictions.

Only one chemical (triclocarban) was highlighted as potentially PMT and PBT, according to the thresholds used to screen M and B properties, i.e., Log Koc < 4 and Log Kow > 4.5, respectively. However, triclocarban classifies as M only when considering the most conservative threshold (Log Koc (3.25 ± 1.07); similarly, the compound has a predicted Log Kow = 4.62 (average among Log Kow = 4.9 (EPI Suite™ v. 4.1) and Log Kow = 4.34 (OPERA v. 2.9), slightly exceeding the screening threshold for B. Further data are necessary to evaluate the B and M behavior of this compound, of which the P and T behavior are officially classified as PT by ECHA [76]. However, according to the Federal Environmental Quality Guidelines, published by Environment Canada in March 2024 [77], this chemical is a PBT, but not a PMT (Log K_OC_ = 4.8). Some differences in the PMT assessment, depending on thresholds applied to assess M behavior, were found for some chemicals, including triclocarban (i.e., citalopram, clopidogrel, desmethylcitalopram, methadone, olanzapine, and triclocarban). These contrasting results, regarding the assessment of B and M properties, demonstrate that, at the screening level, *in silico* models are useful to spot critical areas of the chemical domain where chemicals may have borderline behavior according to predicted properties and to the thresholds applied in the analysis. This is particularly relevant as it shows that predictions within the AD are still associated with uncertainty, which may change the PMT assessment according to specific thresholds. This suggests that, at the screening level, while worst-case assessments based on *in silico* predictions may be the safer solution, experimental data would be necessary to arrive at more realistic assessments. Finally, it is interesting to highlight that, according to additional models used to further characterize the potential PMT and PBT behavior using non-regulatory endpoints listed in Table 4, all the 16 chemicals were also flagged as potential endocrine disruptors.

An important consideration and a key limitation of this study is that the assessment was conducted considering all compounds in the dataset as neutral organic chemicals, due to the lack of adequate QSAR models for IOCs. Furthermore, the current regulatory thresholds used to screen PMTs and PBTs reported in Table 3 apply Log Koc and Log Kow as the reference endpoints at the screening level. This is particularly relevant because properties such as partitioning/distribution and sorption of IOCs can differ significantly from those of the neutral compounds. These properties should be evaluated under an environmentally relevant pH range of four to nine. For example, in the context of B, using Log Kow as a screening threshold is effective for neutral lipophilic substances but less so for dissociated compounds, which may be more rapidly eliminated [78,79,80]. Additionally, Log Kow is not an ideal proxy for bioaccumulation in proteins, potentially overlooking compounds that do not bioaccumulate in lipids [79,81].

In terms of mobility, the sorption behavior of IOCs is influenced by external factors such as pH, water hardness, and the mineral composition of soils or sediments. This is because interactions for charged compounds are primarily driven by IOC speciation and the sorbent’s surface charge, as well as the concentration and composition of other ions in the solution, rather than by hydrophobicity [82]. Thus, the Log Koc for the neutral form is not the most suitable endpoint to evaluate mobility of IOCs. More sophisticated QSARs exist to estimate the sorption of neutral compounds to different sorbents phases and to soils with different compositions [83,84]. However, the application of these models to IOCs is still in a preliminary phase and more sorption data measured under different well-defined chemical conditions in water and soil are needed.

**Figure 5:**
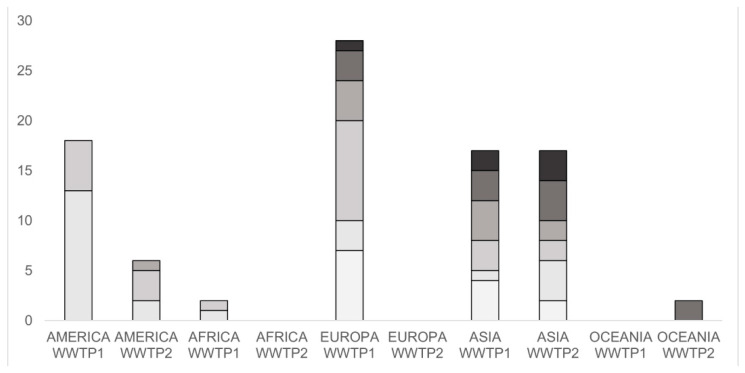
Distribution of RE records of the 16 PMT and PBT compounds in WWTP1 and WWTP2 in different continents. Increasing REs are assigned to 6 color shades, from white to black, in the following order: No Data RE, Other RE, Low RE, Moderate RE, High RE, and Excellent RE.

The combination of the PBT and PMT analysis, reported in Table 7, with the geographical information related to continents where these substances were found in this study (Supplementary Material S1—Table S1), led to the following distribution: Asia 34 records, Europe 28 records, America 24 records, Africa 2 records and Oceania 2 records. Figure 5 shows that, according to the literature, records collected for the 16 PMT and PBT substances are primarily treated in WWTP1 and they are not efficiently removed. In fact, more than half of the reported records in each continent reaches at most a moderate RE. Furthermore, the fact that this profile (i.e., RE Low or RE Other) is found also in records collected in WWTP2 (available in America and Asia) confirms that a minority of these chemicals (i.e., atrazine, chloramphenicol, citalopram, lorazepam, and methadone) persist in the environment independently of the treatment level. This highlights the importance of substituting or regulating worldwide the release of these substances in the aquatic environment, as the water treatment technologies available in different countries, based on the investigated time frame, may not be sufficient to prevent their exposure, and they thus may cause risk due to their potential PMT and PBT behavior to wildlife and humans.

### 3.4. Risk Evaluation

As was described in the methods section, we evaluated the potential risk (PR) for all the studied compounds by dividing relevant environmental concentrations by the relevant toxicity values predicted by QSAR and considering the appropriate assessment factor according to TGD [71]. To this end, as was described in Section 2.4, multiple records of measured concentrations in water, reported in the literature, which were available for the 245 chemicals listed in Supplementary Material S1—Tables S2 and S3, were used as CA and divided by PNEC. PNEC was represented by the highest predicted acute toxicity (i.e., the lowest EC_50_ or LC_50_), quantified across multiple trophic levels (algae, *Daphnia*, and fish), divided by the assessment factor of 1000. Records listed in Supplementary Material S1—Table S2 include 264 PR cases, specifically related to 77 individual substances. Table 8 shows the percentage of PR cases distributed in the five continents versus the respective RE based on information from the literature. Most of these PR cases were found in WWTPs in Europe, followed by America, Asia, Africa, and Oceania. Values reported in Table 8 show that more than 60% of the risk cases are associated with RE data classified as Low or Other. Similar results are reported in Supplementary Material S1—Table S5, where the PR cases are highlighted in a heat map as function of the RE classes in WWTP1 or WWTP2.

On the other hand, about 40% of risk cases were quantified for chemicals with data showing a high RE in WWTPs. This is a remarkable result as it demonstrates that even though the treatment plant may remove most of the substance downstream, the residual concentration could still be sufficiently high to pose potential risks to living organisms. Therefore, efforts should be focused on enhancing treatment efficiencies and reducing upstream inputs to mitigate these risks effectively.

It is interesting to highlight that, according to data published by ECHA in the ECHA Chemicals Database [85] or PubChem [53] (dossier Globally Harmonized System of Classification and Labeling of Chemicals [GHS] [86]), for the 77 chemicals with associated PR, 68 of them have been previously associated with hazards for humans or the environment, while no data are available for the remaining nine substances (i.e., 2-ethylidene-1,5-dimethyl-3,3-diphenylpyrrolidine (EDDP), 2-Phenylbenzimidazole-5-sulfonic acid, brompheniramine, erythromycin-H_2_O, fexofenadine, maprotiline hydrochloride, metamizole, norfluoxetine, and erythromycin). The list of 77 chemicals with a PR and their hazard evaluation, according to the ECHA Chemicals Database or GHS Dossiers, is reported in Supplementary Material S1—Table S6.

Furthermore, seven out of the seventy-seven substances mentioned above (listed in Table 9) were also highlighted among the sixteen chemicals potentially PMT or PBT. Of these substances, PR was identified for 19 records, with 79% found in the effluent of a WWTP1.

## 4. Conclusions

In this paper, we have addressed the identification, according to regulatory criteria, of potential PMT and PBT chemicals or compounds that may pose a risk for the aquatic compartment. This work was performed by combining experimental information describing the RE quantified for 245 PPCPs in WWTPs in different locations worldwide, the related environmental concentrations reported in the literature, and predictions of appropriate endpoints calculated from the molecular structure using multiple *in silico* tools. The analysis of the literature clearly shows a prevalence of studies monitoring the RE of groups of PPCPs, mostly in Europe, Asia, and America, while only a few studies were representative for Africa and Oceania. It is important to highlight that considering the global distribution of PPCPs in WWTPs effluents worldwide, ANTB, OIDM, SHA, PDAM, and AIMB, in addition to AHCV, are the most abundant groups among the 34 analyzed in this study. These classes are also the most resistant to treatment according to RE, independently of the level of treatment (WWTP1 or WWTP2). Other classes of PPCPs, listed in Figure 5, are less efficiently removed by WWTP1 and/or WWTP2. However, these groups of PPCPs are among the least abundant (in terms of number of chemicals), among those found in WWTPs effluents.

Furthermore, it was observed that in most cases, considering 77 chemicals with RE records in both WWTP1 and WWTP2, the RE does not change or improve with the level of treatment moving from WWTP1 to WWTP2. However, there were some exceptions where REs were lower in WWTP2 than in WWTP1. This was associated to RE quantification based on a worst-case approach or, in other cases (e.g., chloramphenicol, diclofenac, lorazepam, methadone, metoprolol, and propranolol), to the possible formation of the chemicals after enhanced treatment, which was documented in the literature. In addition, the fact that some chemicals not efficiently removed in WWTP1 but easily removed in WWTP2 (i.e., clarithromycin, fluoxetine, naproxen, and sulfamethoxazole), were found in Africa, where no information on WWTP2 was available, shows the need to improve WWTP technology in developing countries and/or to substitute those chemicals with less hazardous PPCPs in areas where secondary treatments are not available.

The need to improve the treatment layout is highlighted also for substances which formation after WWTP1 was highlighted in this study and documented in the literature (i.e., lorazepam, methadone, metoprolol, propranolol, methamphetamine, sulpiride and nalidixic acid). This is particularly relevant considering that some of these substances (i.e., lorazepam and methadone) were also highlighted as potentially PMTs according to our screening based on the molecular structure. A total of sixteen substances were highlighted as potential PMTs or PBTs, and which cannot be efficiently removed by water treatment, with the exception made for chloramphenicol and sertraline. However, sertraline is one of the 77 chemicals that were associated to PR according to its environmental concentrations measured in WWTP effluents. Among the 77 chemicals associated with PR, 68 have already been classified as hazardous according to international legislations (i.e., REACH, CLP, and GHS).

Concluding, notwithstanding the fact that further refinement of this study may be conducted in the future on the basis of updated data or to include a more adequate evaluation of PMT and PBT properties of IOCs, based on possible updates in the regulatory endpoints proposed for these assessments, and on the availability of QSARs specific for IOCs, this work demonstrates how the integration of experimental and *in silico* data is useful at the screening level to spot potentially hazardous compounds or possible risk situations. These results provide indications that are useful for scientists and regulators to manage specific chemicals and possible risks by improving WWTP efficiency, reducing the release and/or substituting the problematic chemicals with chemicals that can be more easily removed and do not have PMT and/or PBT behavior as well as those which may cause harmful effects to humans and the environment.

## Figures and Tables

**Table 1:** Levels of treatment, treatment technologies, and final classification of WWTPs were considered in this study.

Level of Treatment	Definition [45] (Treatment Technologies)	WWTP Classification in the Manuscript
Primary	Primary treatments (mechanical treatments) include pre-treatments (such as coarse and fine grids for wastewater screening, oil, and sand removal) and primary clarification whose main goal is solid removal.	WWTP1
Secondary	Secondary treatments are biological treatments (i.e., based on microorganisms-mediated degradation, whose aim is to biologically remove organic matter and nutrients-P and N), such as the oxidation tank and the secondary clarifier. The biological process can be based, for example, on conventional activated sludge process or on MBR (Membrane Biological Reactor) process.	
Tertiary/Advanced	Tertiary treatments include all the treatments aiming at enhancing the removal of nutrients (chemical removal) or to improve the quality of the final effluent by means of chemical-physical processes, advanced oxidation processes (AOPs), disinfection, sand filtration, etc.	WWTP2

**Table 2:** Criteria to assign RE classes.

Removal Class	Criteria
RE Other	RE ≤ 0
RE Low	0 < RE ≤ 50
RE Moderate	50 < RE ≤ 75
RE High	75 < RE ≤ 95
RE Excellent	RE > 95
No RE data	Missing values or below the limit of quantification or detection

**Table 3:** Criteria used in this study for PMT and PBT assessment and to identify potential ED behavior.

Properties	Conclusion	Threshold Value	Reference
Persistence	Potentially P (pP) or very P (pvP)	Biodegrade fast (probability < 0.5) and ultimate biodegradation timeframe (predicted value < 2.25)	[29]
Bioaccumulation for aquatic organisms	Potentially B (pB)	Log Kow > 4.5 Log BCF > 3.3	[29]
	Potentially vB (pvB)	Log BCF > 3.7	[29]
Toxicity (Short-term aquatic toxicity—algae, *Daphnia*, fish)	Potentially T (pT)	EC_50_ or LC_50_ < 0.01 mg/L	[29]
	Potentially T (pT)	EC_50_ or LC_50_ < 0.1 mg/L	[29]
Mobility	Potentially M (pM)	Log Koc < 4 (conservative); Log Koc < 3 (pH range 4–9)	[31,58]
	Potentially vM (pvM)	Log Koc < 3 (conservative); Log Koc < 2 (pH range 4–9)	[31,58]
Endocrine disruption	Potentially endocrine disruptors	Binding to estrogen, androgen receptors and/or to human transthyretin (i.e., thyroid hormone distributor protein)	[59,60,61]

**Table 4:** List of models, endpoints and *in silico* tools used to perform the screening of PMT and PBT chemicals in Supplementary Material S1—Table S3. The screening level refers to the use of regulatory or additional endpoints.

Persistence QSARs	Endpoint	Screening Level	Free Screening Tool
BIOWIN 3	Ultimate Biodegradation probability	L1—Regulatory	QSAR Toolbox 4.4
BIOWIN 6	Fast Biodegradation probability	L1—Regulatory	QSAR Toolbox 4.4
BIOWIN 2	Fast Biodegradation probability	L1—Regulatory	QSAR Toolbox 4.4
Global Half-Life Index (GHLI)	Environmental Fate parameters Global Half-Life Index	L2—Additional	QSAR-ME Profiler v. 1.02
PBT index	Environmental Fate parameters Persistence Bioaccumulation and Toxicity Index	L2—Additional	QSAR-ME Profiler v. 1.02
**Bioaccumulation QSARs**	**Endpoint**	**Screening level**	**Free screening tool**
KOWWIN	Octanol water Partition Coefficient (Log values)	L1—Regulatory	EPI Suite ^TM^ v. 4.1
Log P	Octanol water Partition Coefficient (Log values)	L1—Regulatory	OPERA v.2.9
Log BCF	fish Bioconcentration Factor	L2—Additional	QSAR-ME Profiler v. 1.02
**Mobility QSARs**	**Endpoint**	**Screening level**	**Free screening tool**
KOCWIN from MCI	Organic carbon Partition Coefficient (Log values)	L1—Regulatory	EPI Suite ^TM^ v. 4.1
Soil Koc	Organic carbon Partition Coefficient (Log values)	L1—Regulatory	QSAR-ME Profiler v. 1.02
**Toxicity QSARs**	**Endpoint**	**Screening level**	**Free screening tool**
4.3 *P. promelas* Mod. 1	pLC_50_ (mol/L) fish	L1—Regulatory	QSAR-ME Profiler v. 1.02
4.4 *P. promelas* Mod. 2	pLC_50_ (mol/L) fish	L1—Regulatory	QSAR-ME Profiler v. 1.02
5.3 *O. mykiss*	pLC_50_ (mmol/L) fish	L1—Regulatory	QSAR-ME Profiler v. 1.02
4.5 *P. promelas* Mod. 3	pLC_50_ (mol/L) fish	L1—Regulatory	QSAR-ME Profiler v. 1.02
5.4 *P. promelas* Mod. 4	pLC_50_ (mmol/L) fish	L1—Regulatory	QSAR-ME Profiler v. 1.02
4.1 *P. subcapitata* Mod. 1	pEC_50_ (mol/L) algae	L1—Regulatory	QSAR-ME Profiler v. 1.02
5.1 *P. subcapitata* Mod. 2	pEC_50_ (mmol/L) algae	L1—Regulatory	QSAR-ME Profiler v. 1.02
4.2 *D. magna* Mod. 1	pEC_50_ (mol/L) *Daphnia*	L1—Regulatory	QSAR-ME Profiler v. 1.02
5.2 *D. magna* Mod. 2	pEC_50_ (mmol/L) *Daphnia*	L1—Regulatory	QSAR-ME Profiler v. 1.02

**Table 5:** List of models, endpoints and *in silico* tools used to perform the screening of ED chemicals.

QSAR Model	Endpoint	Screening Level	Free Screening Tool
T4-hTTR	Log RP_ANSA_ [70]	L2—Additional	QSAR-ME Profiler v. 1.02
T4-hTTR	LogRP_FITC-T4_ [70]	L2—Additional	QSAR-ME Profiler v. 1.02
T4-hTTR	LogRP_RLBA_ [70]	L2—Additional	QSAR-ME Profiler v. 1.02
CoMPARA	AR binder [60]	L2—Additional	OPERA v.2.9
CERAPP	ER binder [59]	L2—Additional	OPERA v.2.9

**Table 6:** Percentage distribution of the RE classes for WWTP1 and WWTP2.

Class	WWTP1	WWTP2
RE Other	53 (24%)	15 (14%)
RE Low	50 (22%)	13 (12%)
RE Moderate	28 (13%)	10 (9%)
RE High	24 (11%)	20 (18%)
RE Excellent	32 (14%)	36 (33%)
No RE data	37 (17%)	15 (14%)

**Table 7:** List of the 16 PMT and PBT substances identified using QSAR predictions and the regulatory thresholds reported in Table 2 (Classification is reported in bold). Classification according to thresholds proposed by EU in 2023 [30], is reported in bold, in brackets. Acronyms for PPCPs classes are described in Supplementary Material S2—Table S4.

ID 245	Class	Compound	Classification Based on QSAR (Reg. Endpoints) and Reg. Thresholds ^1^	Classification Based on Additional QSAR	RE WWTP1	RE WWTP2
9	OIDM	2-ethylidene-1,5-dimethyl-3,3-diphenylpyrrolidine (EDDP)	pP or pvP, pB, pT, **PBT**	pB (Log BCF), Strong hTTR Binder	OTHER	-
29	HERB	Atrazine	pP or pvP, vM (M), pT **PMT (PMT)**	Moderate hTTR Binder	---	OTHER
55	ANTB	Chloramphenicol	pP or pvP, pvM (pM), pT **PMT (PMT)**	Moderate hTTR Binder	MODERATE	OTHER
62	PDAM	Citalopram	pP or pvP, npB, pM (notM), pT **PMT (not PMT)**	pPBT (PBTIndex), AR Binder (CoMPARA), Strong hTTR Binder	OTHER	LOW
67	APLM	Clopidogrel	pP or pvP, pM (not pM), pT **PMT (not PMT)**	AR Binder (CoMPARA), Moderate hTTR Binder	LOW	---
79	PDAM	Desmethylcitalopram	pP or pvP, pM (not pM), pT **PMT (not PMT)**	AR Binder. (CoMPARA) Moderate hTTR Binder	OTHER	---
127	SHA	Lorazepam	pP or pvP, pvM (pM), pT **PMT (PMT)**	AR Binder, (CoMPARA) Weak hTTR Binder	OTHER	LOW
128	PDAM	Lormetazepam	pP or pvP, pvM (pM), pT **PMT (PMT)**	pPBT (PBTIndex), Strong hTTR Binder	LOW	---
139	OIDM	Methadone	pP or pvP, pM (not Pm), pT **PMT (not PMT)**	AR Binder (CoMPARA), Moderate hTTR Binder	OTHER	LOW
147	ANTB	Miconazole	pP or pvP, pB, pT **PBT**	pP(GHLI), pPBT (PBTIndex), AR Binder (CoMPARA), Strong hTTR Binder	LOW	---
154	AHCV	Nafronyl	pP or pvP, pB, pT **PBT**	AR Binder (CoMPARA, Moderate hTTR Binder	LOW	---
171	PDAM	Norsertraline	pP or pvP, pB, pT **PBT**	pPBT (PBTIndex), Strong hTTR Binder	OTHER	---
176	PDAM	Olanzapine	pP or pvP, pM (not pM), pT **PMT (not PMT)**	Moderate hTTR Binder	NO DATA	---
205	PDAM	Sertraline	pP or pvP, pB, pT **PBT**	pP (GHLI), pPBT (PBTIndex), pB (Log BCF), AR Binder (CoMPARA, Strong hTTR binder	OTHER	HIGH
210	ANTB	Sulfachloropyridazine	pP or pvP, pvM (pM), pT **PMT (PMT)**	Strong hTTR Binder	NO DATA	---
235	ANTB	Triclocarban	pP or pvP, pB, pM (not pM), pT **PBT and PMT (not PMT)**	AR Binder (CoMPARA). Strong hTTR Binder	LOW	---

^1^ QSAR predictions: potentially P (pP) or very P (pvP); potentially B (pB) or potentially vB (pvB); potentially M (pM) or potentially vM (pvM); potentially T (pT). Experimental data: mobile (M); bioaccumulable (B).

**Table 8:** Percentage of PR cases distributed in the five continents versus the respective removal efficiency (RE) based on information from the literature. The last column represents the total number of chemicals in each continent, while the last row shows the total number of chemicals in each class across the five continents.

	Re Low		Re Other		Re Moderate		Re High		Re Excellent		Total
	n°	%	n°	%	n°	%	n°	%	n°	%	n°
AMERICA	18	21.18%	3	3.33%	7	13.21%	5	15.63%	0	0.00%	33
AFRICA	9	10.59%	1	1.11%	3	5.66%	5	15.63%	0	0.00%	18
EUROPE	51	60.00%	66	73.33%	32	60.38%	19	59.38%	1	25.00%	169
ASIA	7	8.24%	18	20.00%	10	18.87%	1	3.13%	3	75.00%	39
OCEANIA	0	0.00%	2	2.22%	1	1.89%	2	6.25%	0	0.00%	5
Total	85	32.20%	90	34.09%	53	20.08%	32	12.12%	4	1.52%	264

**Table 9:** List of potential PBT and PMT substances with indication of potential risk and information reported in the ECHA Chemicals Database [85] or PubChem [53].

ID 245	Compound	Classification Based on QSAR	PMT and PBT Assessment	Literature Information
9	2-ethylidene-1,5-dimethyl-3,3-diphenylpyrrolidine (EDDP)	pP or pvP, pB, pT,	PBT	No Classification
62	Citalopram	pP or pvP, npB, pM, pT	PMT	Health hazard|Environmental hazard (Pubchem)
127	Lorazepam	pP or pvP, nB, pvM, pT	PMT	Health hazard|Reproductive toxicity|Environmental hazard (Pubchem)
128	Lormetazepam	pP or pvP, npB, pvM, pT	PMT	Health hazard|Reproductive toxicity (Pubchem)
139	Methadone	pP or pvP, nB, pM, pT	PMT	Health hazard|STOT-SE|Environmental hazard (ECHA)
205	Sertraline	pP or pvP, pB, npM, T	PBT	Health hazard|Environmental hazard (Pubchem)

## Data Availability

The original contributions presented in this study are included in the article/Supplementary Material. Further inquiries can be directed to the corresponding authors.

## Supplementary Materials

The following supporting information can be downloaded at: https://www.mdpi.com/article/10.3390/toxics13010006/s1, Supplementary Material_S1; Supplementary Material_S2. Supplementary Material_S1 (Table S1: information on water treatment plants and the related literature (76 WWTPs); Table S2: chemical identifiers, quantification of the RE and PR (2034 records); Table S3: chemical identifiers, QSAR predictions (245 chemicals); Table S4: comparison of RE data in WWTP1 and WWTP2 (77 chemicals); Table S5: heat map (distribution of molecules with potential risk (country, removal and treatment plant)); Table S6: hazard evaluation (77 chemicals)). Supplementary Material_S2 (Table S1: Values of REs used in specific situations; Table S2: QI Scores; Table S3: Classes of molecules and number of molecules and records per class; Table S4: Description and meaning of acronyms of chemical substance classes; Table S5: Additional details about the exceptions; Figure S1: Classes of molecules and related percentages of molecules and records).
