# Supplementary material for: Global Assessment of Emerging Contaminant Removal in Wastewater Treatment Plants: In Silico Hazard Screening and Risk Evaluation
Source: Toxics. 2024 Dec 25;13(1):6. doi: 10.3390/toxics13010006 (PMC11768813; doi:10.3390/toxics13010006)
Supplement: Supplementary file 1 [file toxics-13-00006-s001.zip › Revised_S2_Rev_Final.pdf]

Supplementary Information for the manuscript

Global Assessment of Emerging Contaminant Removal in Wastewater Treatment Plants: *In Silico* Hazard Screening and Risk Evaluation

Arianna Sgariboldi [1,2], Elena Posté [1,3], Nicola Chirico [1], Alessandro Sangion [4], Marco Evangelista [1,2], Cristiana Morosini [2], Andrea Re [1,5], Vincenzo Torretta [1] and Ester Papa [1,*]

[1]Department of Theoretical and Applied Sciences, University of Insubria, via J.H. Dunant 3, 21100 Varese, Italy; asgariboldi@uninsubria.it; elena.poste@aircleansrl.com; nicola.chirico@uninsubria.it; mevangelista@uninsubria.it; reandrea.96@gmail.com; vincenzo.torretta@uninsubria.it; ester.papa@uninsubria.it
[2] Department of Science and High Technology, University of Insubria, via Valleggio 11, 22100 Como, Italy; asgariboldi@uninsubria.it; mevangelista@uninsubria.it; cristiana.morosini@uninsubria.it;
[3] AIR CLEAN S.r.l., via Trento 37, 20017 Rho, Italy; elena.poste@aircleansrl.com
[4] ARC Arnot Research & Consulting, Toronto, Canada; alessandro@arnotresearch.com
[5] Xylem Water Solutions Italia S.r.l., Via G. Rossini, 1/A, 20045 Lainate, Italy; reandrea.96@gmail.com

e-mail: *ester.papa@uninsubria.it*,

Definition of the Quality Index

For each data or article included in the study, a quality score was assigned on the basis of multiple indexes. The indexes are listed hereafter:

- **Molecule identification index**: it considers if a molecule was clearly identified considering Name, and CAS number. Score 1 was assigned to molecules identified with certainty, otherwise score 0 was assigned.
- **RE data index**: it considers the origin of RE data. Score 1 was assigned to RE provided by the authors in the literature, score 0 was assigned to RE calculated as described in chapter “2.2.2 Removal efficiency (RE)”.
- **General information index:** it ranges from 0 to 4. The final value of this index is given by the sum of four sub-indexes that compose it:
    - Hydraulic Retention Time - HRT: score 1 was assigned to studies that considered the HRT during the sample campaign, otherwise score 0 was assigned.
    - Sample: score 1 was assigned to composite samples, score 0 was assigned to grab samples or to not clear samples (i.e., missing details about the sampling procedure).
    - Sorption on sludge: score 1 was assigned to data from articles where sorption on sludge was considered in the calculation of the overall RE, otherwise score 0 was assigned.
    - WWTPs description: score 1 was assigned to data from articles where the WWTPs were described properly (list of all the steps or, at least, of the principal steps), otherwise score 0 was assigned.

Table S1. Values of REs used in specific situations. <LOQ or <LOD were reported when concentrations used to calculate RE were below the limit of quantification or the limit of detection, respectively.

**Influent sample**	**Effluent sample**	**RE adopted**
Concentration >0	<LOQ	100%
Concentration >0	n.d.*	100%
<LOQ	Concentration >0	-100%
n.d.	Concentration >0	-100%
<LOQ	n.d.	<LOD
n.d.	<LOQ	<LOQ
<LOD	<LOD	<LOD
<LOQ	<LOQ	<LOQ
n.d.	n.d.	n.d.

*n.d. = not detected

RE equal to 100% was adopted if a concentration >0 was found in the influent and the concentration of the substance was below the limit of quantification in the effluent or if the substance was not detected in the effluent samples. Similarly, the value of -100% was adopted if the substance was not detected in the influent samples or if the concentration in the influent was below the limit of quantification, and a concentration >0 was found in the effluent

Combination of multiple indices to create the Quality Index

The results of the assignments of the scores of the Quality indices are summarized in Table 1 and described as follows:

Molecule identification index - 1999 records have score 1 and 35 records have score 0. This means that almost all the molecules were correctly identified.

RE data index: 749 records have score 1 while 1285 records have score 0. This means that most REs were not provided by the authors of the papers, so their calculation was necessary.

General information index: 18 records have score 4, 683 records have score 3, 693 records have score 2, 360 records have score 1 and 280 records have score 0. These results derive from the four subindexes HRT, type of samples, sorption to sludge, and description of WWTPs, which were used to describe different levels of information in the 32 articles listed in Online Source S1 – Table S1

- HRT: 10 papers have score 1 and 22 articles have score 0. This means that the two thirds of the studies did not consider the HRT during the sampling campaigns.
    - type of samples: 22 articles have score 1 and 10 have score 0. This means that two thirds of the studies used samples more reliable than grab samples.
- sorption to sludge: 5 articles have score 1 and 27 have score 0. This means that most studies did not consider if the REs were due to degradation or to the transfer of a compound from the liquid phase to the solid phase.
- description of WWTPs: 26 articles have score 1 and 6 papers have score 0. This means that most WWTPs were described properly.

Table S2. QI Scores

Index	Score 0	Score 1	Score 2	Score 3	Score 4
**Molecule identificatio mn index ***	35	1999	-	-	-
**Origin of RE data index***	1285	749	-	-	-
**General info index***	280	360	693	683	18

HRT°	22	10	-	-	-
Type of Sample°	10	22	-	-	-
Sorption to sludge°	27	5	-	-	-
WWTPs description°	6	26	-	-	-

*Index referred to the experimental data

°Index referred to the paper

The sum of the scores of the individual indices listed in table S2, led to the Quality Index (QI). This index was used to assign original data points to four quality-classes assigned as follows:

- score 0-1 = Quality Index Low (QI Low);
- score 2-3 = Quality Index Moderate (QI Moderate);
- score 4-5 = Quality Index Good (QI Good);
- score 6 = Quality Index Excellent (QI Excellent);

Out of the original 2034 records collected in Online Source S1 - Table S2, 18 records were identified as QI Excellent, 794 as QI Good, 940 as QI Moderate and 282 as QI Low. This means that only the 13.9% of the whole data have a low quality. For this reason, these data were not discarded from further analysis.

Table S3. Classes of molecules and number of molecules and records per class

CLASS	N° records per class	%	N° molecules /compounds per class	%
Antibiotics	531	30.31%	47	19.18%
Analgesics/anti-inflammatories and metabolites	307	17.52%	26	10.61%
Antihypertensives and cardiovascular drugs	139	7.93%	24	9.80%
Psychiatric drugs/antidepressants and metabolites	138	7.88%	26	10.61%
Lipid regulators	134	7.65%	7	2.86%
Opioids, illicit drugs and metabolites	82	4.68%	32	13.06%
Antiepileptics and metabolites	71	4.05%	4	1.63%
Stimulants and metabolites	53	3.03%	4	1.63%
Hormones and steroids	45	2.57%	9	3.67%
Sedatives-hypnotics-anxiolytics	40	2.28%	8	3.27%
Antihistamines and metabolites	30	2.11%	9	4.08%
Disinfectants	24	1.37%	1	0.41%
H2 receptor agonists	21	0.80%	3	0.82%
UV filters	20	1.14%	6	2.45%
Repellent	14	0.80%	2	0.82%
Endocrine disruptors	13	0.74%	1	0.41%
Fragrances	12	0.68%	3	1.22%
Human indicators and metabolites	9	0.51%	2	0.82%
Diuretics	9	0.51%	1	0.41%
Parabens	8	0.46%	4	1.63%
Surfactants	8	0.46%	3	1.22%
Antiplatelet and metabolites	8	0.46%	2	0.82%
Anti-cancer	7	0.40%	5	2.04%
Antidiabetics	6	0.34%	2	0.82%
Phosphodiesterase type V inhibitors	4	0.23%	1	0.41%
Anesthetics and metabolite	3	0.17%	3	1.22%
Corticosteroids	3	0.17%	3	1.22%

Anticoagulant	3	0.17%	1	0.41%
Asthma medication	3	0.17%	1	0.41%
Herbicide	2	0.11%	1	0.41%
X-ray contrast media	2	0.11%	1	0.41%
Cough suppressant	1	0.06%	1	0.41%
Other	1	0.06%	1	0.41%
Veterinary	1	0.06%	1	0.41%

Table S4. List of acronyms used for different Pharmaceutical and Personal Care Products (PPCPs) classes.

Acronym	Class
AIMB	Analgesics/anti-inflammatories and metabolites
ANME	Anesthetics and metabolite
ANTB	Antibiotics
ACNC	Anti-cancer
ANTC	Anticoagulant
ADBT	Antidiabetics
AEME	Antiepileptics and metabolites
AHME	Antihistamines and metabolites
AHCV	Antihypertensives and cardiovascular drugs
APLM	Antiplatelet and metabolites
ASTM	Asthma medication
CORT	Corticosteroids
COSU	Cough suppressant
DISF	Disinfectants
DIUR	Diuretics
ENDO	Endocrine disruptors
FRAG	Fragrances
H2RA	H2 receptor agonists
HERB	Herbicide
HORM	Hormones and steroids
HIMB	Human indicators and metabolites
LIRE	Lipid regulators
OIDM	Opioids, illicit drugs and metabolites
OTHR	Other
PARB	Parabens
PDI-V	Phosphodiesterase type V inhibitors
PDAM	Psychiatric drugs/antidepressants and metabolites
REPL	Repellent
SHA	Sedatives-hypnotics-anxiolytics
STME	Stimulants and metabolites
SURF	Surfactants
UVFL	UV filters
VET	Veterinary
XCM	X-ray contrast media

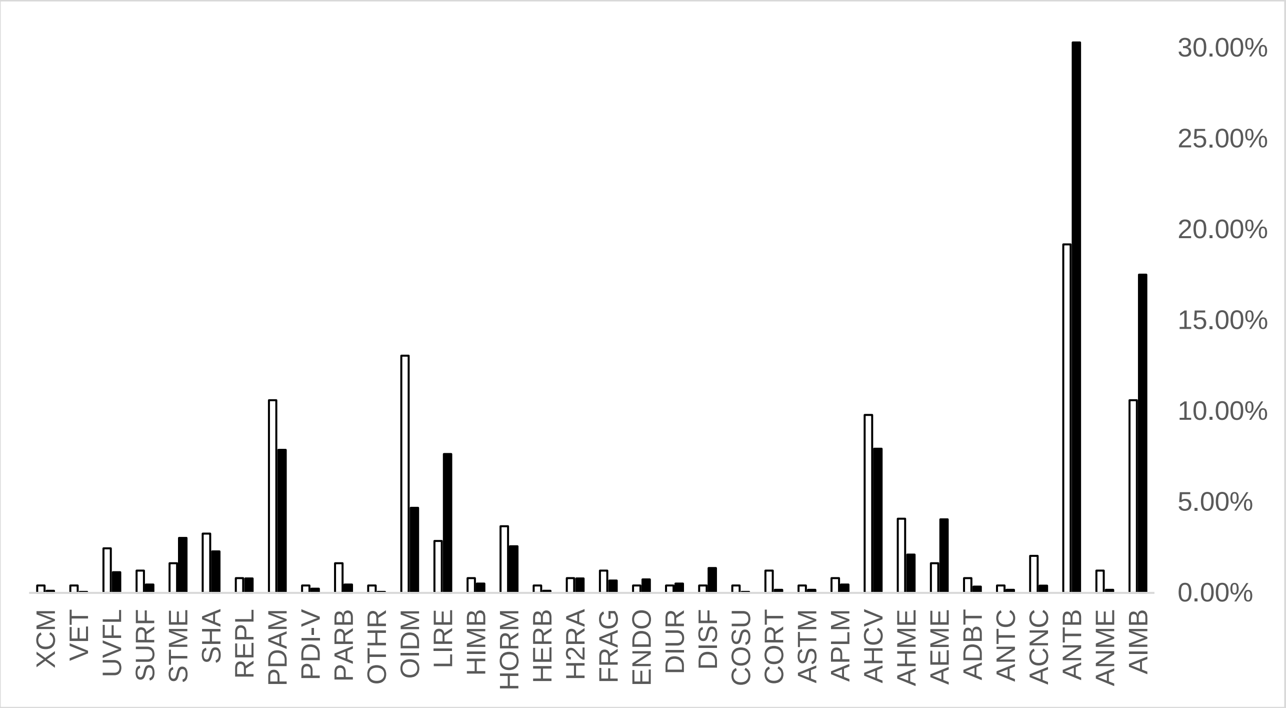

Fig. S1 – Classes of molecules and related percentages of molecules and records (% of molecules white bars, % of records = black bars)

Table S5. Additional details about the exceptions

	WWTP1	WWTP2
Diphenhydramine	RE OTHER x 1record	RE OTHER x 2 Records
	RE MODERATE x 3 Records	
	RE LOW x 1 Record	
	**RE MODERATE**	**RE OTHER**
Ampicillin	RE HIGH x 5 records	RE no data x 8 records
	RE LOW x 1 record	RE OTHER x 1 record
		RE MODERATE x 1 record
		RE HIGH x 1 record
		RE EXCELLENT x 1 record
	**RE HIGH**	**RE OTHER**
Sulfamethoxazole	RE OTHER x 6 records	RE OTHER X 9 records
	RE LOW x 5 records	RE MODERATE X 4 records
	RE MODERATE x 9 records	RE HIGH x 8 records
	RE HIGH x 3records	RE EXCELLENT x 6 records
	RE EXCELLENT x 2 records	
	RE no data x 1 records	
	**RE MODERATE**	**RE OTHER**
Alprazolam	RE OTHER x 1 record	RE OTHER x 1 record
	RE LOW x 2 records	
	RE no data x 1 record	
	**RE LOW**	**RE OTHER**
Sotatol	RE LOW x 1 record	RE OTHER x 1 record
		RE LOW x 1 record
	**RE LOW**	**RE OTHER**
Diazepam	RE OTHER x 2 records	RE OTHER x 1 record
	RE LOW x 4 records	
	RE MODERATE x 2 records	
	RE HIGH x 1 record	
	**RE LOW**	**RE OTHER**
Sulfapyridine	RE MODERATE x 1 record	RE LOW x 1 record
	**RE MODERATE**	**RE LOW**
Citalopram	RE OTHER x 5 records	RE LOW x 1 record
	RE LOW x 5 records	

	RE MODERATE x 1 record	
	RE HIGH x 1 record	
	**RE OTHER**	**RE LOW**
Temazepam	RE OTHER x 1 record	RE LOW x 1 record
	**RE OTHER**	**RE LOW**
Chloramphenicol	RE HIGH x 2 records	RE OTHER x 4 records
	RE MODERATE x 2 records	RE LOW x 2 records
	RE EXCELLENT x 1 records	RE HIGH x 4 records
		RE MODERATE x 2 records
		RE EXCELLENT x 3 records
		RE no data per 2 records
	**RE MODERATE**	**RE OTHER**
